# A bacterial size law revealed by a coarse-grained model of cell physiology

**DOI:** 10.1371/journal.pcbi.1008245

**Published:** 2020-09-28

**Authors:** François Bertaux, Julius von Kügelgen, Samuel Marguerat, Vahid Shahrezaei

**Affiliations:** 1 Department of Mathematics, Imperial College London, London, United Kingdom; 2 MRC London Institute of Medical Sciences (LMS), London, United Kingdom; 3 Institute of Clinical Sciences (ICS), Faculty of Medicine, Imperial College London, London, United Kingdom; 4 Institut Pasteur, USR 3756 IP CNRS, Paris, France; University of Pittsburgh, UNITED STATES

## Abstract

Universal observations in Biology are sometimes described as “laws”. In *E*. *coli*, experimental studies performed over the past six decades have revealed major growth laws relating ribosomal mass fraction and cell size to the growth rate. Because they formalize complex emerging principles in biology, growth laws have been instrumental in shaping our understanding of bacterial physiology. Here, we discovered a novel size law that connects cell size to the inverse of the metabolic proteome mass fraction and the active fraction of ribosomes. We used a simple whole-cell coarse-grained model of cell physiology that combines the proteome allocation theory and the structural model of cell division. This integrated model captures all available experimental data connecting the cell proteome composition, ribosome activity, division size and growth rate in response to nutrient quality, antibiotic treatment and increased protein burden. Finally, a stochastic extension of the model explains non-trivial correlations observed in single cell experiments including the adder principle. This work provides a simple and robust theoretical framework for studying the fundamental principles of cell size determination in unicellular organisms.

## Author summary

Bacteria respond to environmental changes by adjusting their molecular composition, cell size and growth rate. This plasticity is thought to result from years of evolution and to be at least in part optimal for bacterial physiology. Over the past decades, quantitative studies of bacterial growth have revealed simple phenomenological relationships, called “growth laws”, which link cell size and cell composition to the growth rate. Simplified mathematical models of cell physiology are useful tools to gain quantitative understanding of the molecular mechanisms that underlie growth laws. For instance, these models helped explaining how optimal allocation of cellular resource to physiological processes and pathways governs the cell molecular composition in response to specific environmental conditions. In this study, we have extended and integrated existing mathematical models and used experimental data from several recent studies to understand the co-regulation of cell composition, cell size and the cellular growth rate. The model predictions uncovered a novel “size law” that links cell size to the levels of metabolic proteins and the fraction of active ribosomes present in the cell. This work provides a useful theoretical tool and a quantitative basis for understanding mechanistically bacterial physiology as a function of external conditions.

## Introduction

The behavior of complex biological systems can be described by surprisingly simple rules or “laws”, which connect quantitatively aspects of the cell composition with its physiology. Over the last six decades, the discovery of several rules, called the bacterial growth laws, transformed the field of microbial physiology [1–8]. Growth laws describe relationships between the exponential growth rate and cellular parameters. One such law states that the proportion of the cellular content dedicated to ribosomes is linearly dependent on the growth rate (we call this hereafter the first growth law). A second growth law states that average cell size is an exponential function of the growth rate (referred to hereafter as the second growth law). These laws formalize emerging relations between cellular processes quantitatively and therefore generate testable hypothesis for mechanistic studies of the molecular circuitry that underpin cell physiology [9,10].

What are the biological principles underlying the first growth law? The law was first rationalized as a direct consequence of the catalytic role of ribosomes in protein synthesis [11]. Indeed, if every ribosome in a cell synthesises proteins at a constant condition-independent rate, then the steady-state growth rate will be proportional to the fraction of cellular content devoted to ribosomes. However, this does not explain what sets the amount of resources invested into ribosomes in a given environment, and what prevents the cell to increase this investment in order to grow faster. Theoretical and experimental studies identified cellular resource allocation as a key concept to address these questions [12]. The theory of resource allocation describes how different groups of proteins with similar function (proteome sectors) are regulated reciprocally in conditions that affect growth rate. In particular, these studies showed that cells invest higher amounts into ribosomes when nutrient quality increases because the investment in metabolic proteins required to achieve a given growth rate is reduced. This explains the positive correlation of the proteome fraction dedicated to ribosomal proteins with the growth rate and provides a model for how the fraction of ribosomes is set. Interestingly, Hwa and co-workers [13] also showed that the first growth law is not always valid when growth rate is not modulated by nutrient quality. While the law remains valid when useless proteins are over-expressed, it breaks down when translation is inhibited. Based on these findings, they proposed a phenomenological model of proteome allocation whose predictions go beyond the first growth law because it captures these orthogonal types of growth rate modulation [13]. More mechanistic coarse-grained models predicting both the growth rate and the coarse-grained proteome as a function of growth conditions have also been proposed confirming and extending these findings [14–25].

In contrast, the mechanisms underlying the second growth law, which connects growth rate and cell size, are less well understood. In 1968, Donachie [26] proposed that, in bacteria, an exponential dependency of cell size on growth rate indicates that: 1) the timing of DNA replication is fully controlled by cell size, and is triggered at a constant volume per origin of replication; 2) cell division is enslaved to the DNA replication processes, and occurs after a constant time-interval following replication initiation, which encompasses DNA replication and cell division (*C+D* period). However, while recent work has confirmed that DNA replication is strongly size-controlled [27] and that the initiation size per origin is invariant across a large range of growth rates and types of growth rate modulations [28], what determines the duration of the *C+D* period is still unclear. Moreover, the relationship between cell size and growth rate does not follow the second growth law, when useless proteins are over-expressed on gene expression is increased or translation is inhibited [28,29]. This indicates that duration of the *C+D* period is regulated in non-trivial ways outside of the canonical growth modulations based on nutrient quality. To date, unlike for the first growth law, mechanistic understanding of this phenomenon in connection to the second growth law is lacking.

The two growth laws discussed so far are based on average cell population measurements. However, the variations at the single cell level from the population average also provides rich and complementary insights into the biology of cell growth and division [30,31]. Cells can control their size through a ‘sizer’ mechanism, where regardless of birth size they grow to an average size and then they divide. But, recent advances in single-cell techniques led to the discovery that bacterial cells achieve cell size homeostasis via an ‘adder’ principle, where cells add a constant size between birth and division independent of the birth size [32,30,33]. The adder phenomenon has been observed in several bacteria, yeast, archea and mammalian cells [34,30,35–42]. Despite its broad conservation, the mechanistic basis of this phenomenon remains elusive and hotly debated. It has been suggested that the adder phenomenon is related to second growth law assuming that replication controls cell division [43,44]. However, this model is challenged by recent studies that questioned the role of DNA replication in controlling division of single cells [45–47].

Here, we present a coarse-grained model of bacterial physiology that unifies the proteome allocation theory with the structural model of division control [29,30,48]. Building on existing works we have developed a simple, quantitative and actionable model able to integrate processes which so far had only been modelled in isolation. Specifically, this model relies on a minimal number of fitting parameters to capture experimental measurements of proteome allocation, ribosome activity and cell size across several growth modulations. It allowed to formalise a new bacterial “size law” which states that cell size depends on the metabolism proteome sector mass fraction and the fraction of active ribosomes. This law is simple and more general than the original second growth law. Finally, the model exhibits single cell properties that are consistent with experimental observations including the adder size control.

## Results

### A minimalistic whole-cell coarse-grained model to predict cell composition, growth rate and cell size

We developed a minimalistic whole-cell coarse-grained mathematical model to predict cell composition, growth rate and cell size for at least three types of growth rate modulation: 1) change of nutrient quality, 2) translation inhibition by chloramphenicol and 3) over-expression of useless proteins. These modulations were selected because they have been instrumental in uncovering key principles of proteome allocation underpinning the first growth law [13] and have abundant experimental measurements available [13,29,30,49,28]. Our model extends the existing whole-cell coarse-grained models [14–25] by including for the first time both cell composition and cell size. In addition, our model captures a wide-range of experimental measurements including cell size [29–30], proteome allocation [13,49] and ribosome activity [49] data.

**Fig 1:**
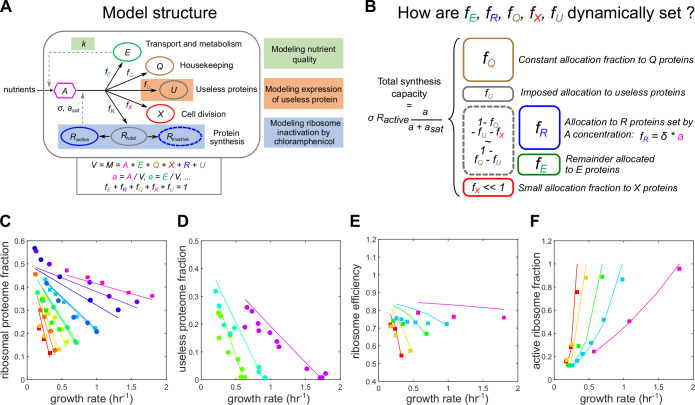
A simple whole-cell coarse-grained model of bacterial growth reproduces proteome allocation and ribosome activity data. (A) Schematic of model structure displaying model components (proteome sectors E, R, Q and X and protein precursor A) and reactions (metabolism, protein synthesis). How the three types of growth rate modulation (nutrient quality, expression of useless protein and ribosome inactivation) are modeled is also highlighted. The rate of precursor creation is proportional to the amount of metabolic enzymes E. The total protein synthesis rate is proportional to the number of active ribosomes, and the synthesis rate per ribosome (the ribosome efficiency) is dependent on the precursor concentration via a Michaelis-Menten relationship. A full description of the model is given in the Methods section. (B) Allocation of total protein synthesis capacity between proteome sectors. A fixed, condition-independent fraction *f*_*Q*_ is allocated to housekeeping proteins. Expression of useless protein imposes a fixed allocation *f*_*U*_. The allocation to R proteins is proportional to precursor concentration, and the remainder is allocated to E proteins (assuming that *f*_*X*_≪1). (C-F) Model predictions (solid lines) agree with experimental data (circles–Scott et al. 2010 [13], squares–Dai et al. 2017 [49]). Parameters were all fixed from published data (see *Methods*). (C) Ribosomal proteome fraction data for nutrient and chloramphenicol growth rate modulations. Colors indicate different nutrient qualities. (D) Relationship between growth rate and useless proteome fraction for forced expression of useless protein. (E-F) Ribosome efficiency ($\frac{a}{a+a_{sat}}$) and active ribosome fraction ($\frac{R_{a}}{R}$).

As in previous work, the model (Fig 1, see *Methods* for a mathematical description) considers only two types of molecular components: protein precursors (noted *A*) and proteins [15,16,20]. Proteins are split between four different classes (or sectors): transport and metabolism proteins (referred to as only metabolic sector for brevity) (*E*), ribosomal proteins (*R*), housekeeping proteins (*Q*), and division proteins (*X)* [29,30]. *E* proteins catalyse the import and transformation of external nutrients (not explicitly modelled) into precursors *A*. *R* proteins are involved in the synthesis of proteins from precursors. *Q* proteins are involved in cellular functions that do not directly contribute to growth. *X* proteins are regulating cell division (see below). Lastly, when growth rate is modulated by over-expression of useless proteins, we consider an additional class of “useless” proteins noted *U* (Fig 1). All proteins are stable in our model and only diluted by cell growth. The model enforces mass conservation—cell mass being defined as the sum of all molecular components. Finally, except for the import and transformation of nutrients into precursors, all reactions leave cell mass unchanged and the model assumes mass density homeostasis (cell volume and cell mass are therefore equivalent, and so are mass fractions and concentrations), as observed experimentally [29].

To fully specify the model, one should describe how the protein synthesis allocation fractions (how much of the total protein synthesis capacity is devoted to each protein class) are set (Fig 1B). A fixed, condition-independent fraction *f*_*Q*_ is allocated to *Q* proteins. We assume that the allocation fraction to division proteins is small (*f*_*X*_≪1). The allocation fraction *f*_*U*_ is imposed and represents the level of useless protein expression. The main allocation decision is therefore between *f*_*R*_ and *f*_*E*_. In this study, rather than assuming growth rate maximization to set these fractions, we assume that *f*_*R*_ (and hence *f*_*E*_) is dynamically regulated as detailed below. This choice is motivated by the recent observation [49] that the ribosome translation elongation rate exhibits a Michaelis-Menten dependence on the total ribosome proteome fraction across nutrient and chloramphenicol-mediated growth modulations. This implies that addition of chloramphenicol to cells growing in poor media results in an increase in the translation elongation rate. This observation is somewhat unintuitive and had not been captured by previous modelling studies [50,51]. Interestingly, this dependency can be simply reproduced in our model by assuming that the allocation fraction to ribosomal proteins *f*_*R*_ is proportional to the precursor concentration *a*, *f*_*R*_ = *δa* (Methods). We further assume that *δ* is large enough to ensure that *a*≪1, as the steady-state concentration of free amino-acids is known to remain small [10]. Simulations show that this approximation is already valid when *δ* = 5 (Fig A in S1 Text). Then, the steady-state cell composition and growth rate can be predicted and depend only on: 1) four condition-independent parameters: the maximal ribosome speed *σ*, the ribosome regulation constant *K*, the housekeeping protein allocation fraction *f*_*Q*_ and the unbinding constant $k_{off}^{cm}$ of chloramphenicol-ribosome complexes; and 2) three growth modulation parameters: the medium nutrient quality *k*, the chloramphenicol-imposed inactivation rate of ribosomes $k_{on}^{cm}$, and the useless protein allocation fraction *f*_*U*_.

The model reproduces quantitatively proteome allocation data [13,49] and ribosome activity data [49] for the three types of growth modulation (Fig 1C–1F). This agreement is obtained without parameter fitting, because the parameters *σ*, *K*, *f*_*Q*_ and $k_{off}^{cm}$ are directly constrained from experimental measurements [13,49]. To our knowledge, this is the first coarse-grained model of bacterial growth to reproduce both proteome allocation and ribosome activity data.

While it is often assumed that proteome fractions are optimally allocated to maximize growth rate [15], in our model proteome fractions are dynamically set by the simple regulation *f*_*R*_ = *δa*. Interestingly, this regulation achieves near-optimal growth rates across the three types of growth rate modulations considered in this study (Fig B in S1 Text). This is consistent with previous modelling work that shows at fast growth bacterial allocation of ribosome fractions is tuned to approximate growth-rate maximisation [18]. However, we note that the model assuming optimal proteome allocation does not reproduce the ribosome activity data of Fig 1E.

**Fig 2:**
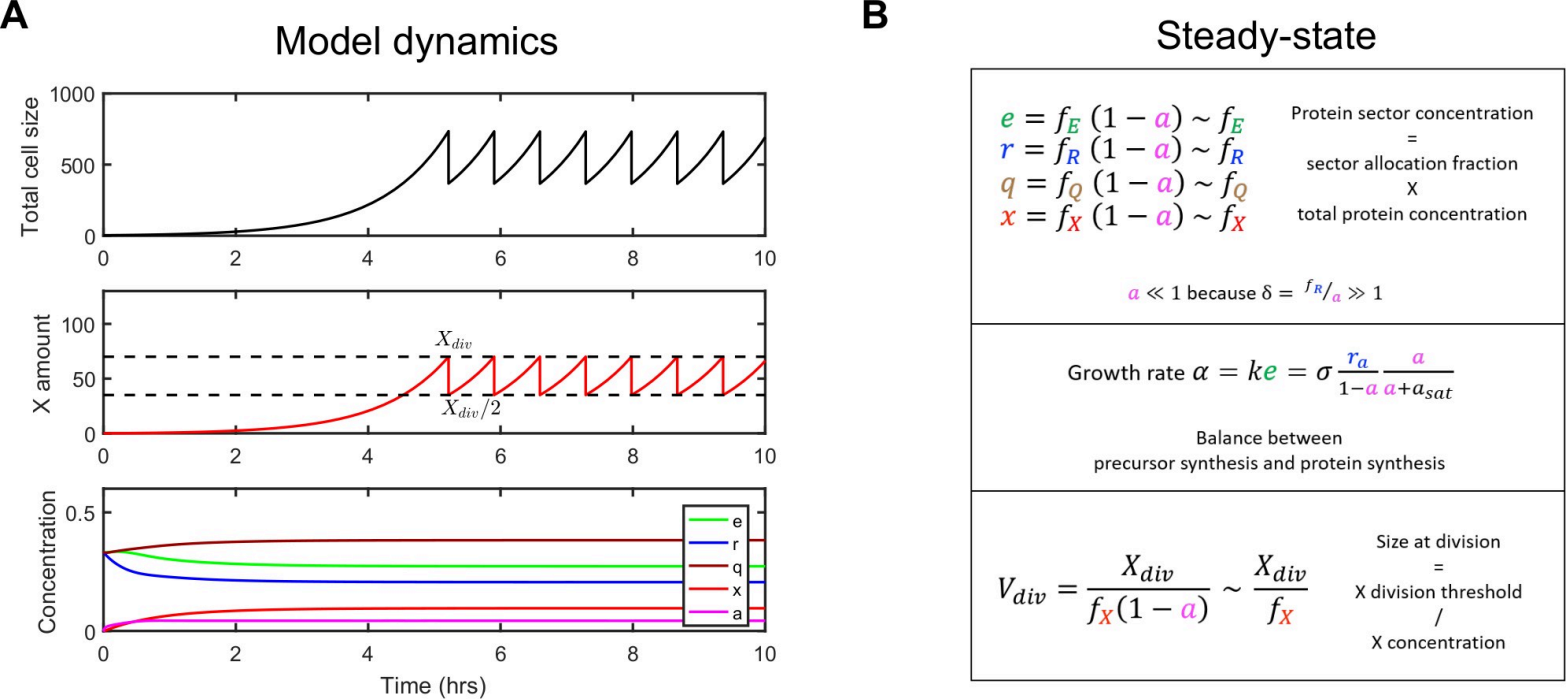
Integration of the structural model enables prediction of both cell composition and cell size. (A) Simulation of model dynamics. After cell division (triggered when *X* number reaches *X*_*div*_), cell content is partitioned equally among daughter cells and only one is followed ('mother machine' setting, Wang et al., 2010). After a transient adaptation period, concentrations of cellular components are constant, cellular growth (size increase) is exponential and division occurs at a constant size. Here, nutrient quality is such that the growth rate is 1 hr^−1^ and there is no chloramphenicol nor useless expression. (B) Equations characterizing model steady-state together with their intuitive interpretation (see *Methods* for their derivation). *r*_*a*_ denotes the concentration of active ribosomes.

Our model also explicitly includes cell division and assumes that it is triggered by the accumulation of proteins belonging to the *X* sector to an absolute copy number threshold *X*_*div*_ (Fig 2A), as proposed before in the structural model [48,30,29]. This design enables prediction of changes in both cell size and coarse-grained cell composition as a function of cell growth. Note that for simplicity and because it appears unnecessary, we do not assume that *X* proteins are destroyed immediately after division, in contrast to other studies [30,48]. Altogether, our model assumptions result in simple and intuitive equations characterizing model steady-states (Fig 2B). Importantly, steady-state concentrations of cell components are not dependent on the cell cycle as expected under balanced growth and are valid when cell division is not modeled (as done for the fitting of the proteome fraction data above).

In summary, we have developed a whole-cell model of cell physiology that captures changes in proteome fractions and ribosome activity as a function of growth conditions while uniquely incorporating direct modeling of cell division control.

### Cell size can be predicted from coarse-grained cell composition across all conditions revealing a novel size law

**Fig 3:**
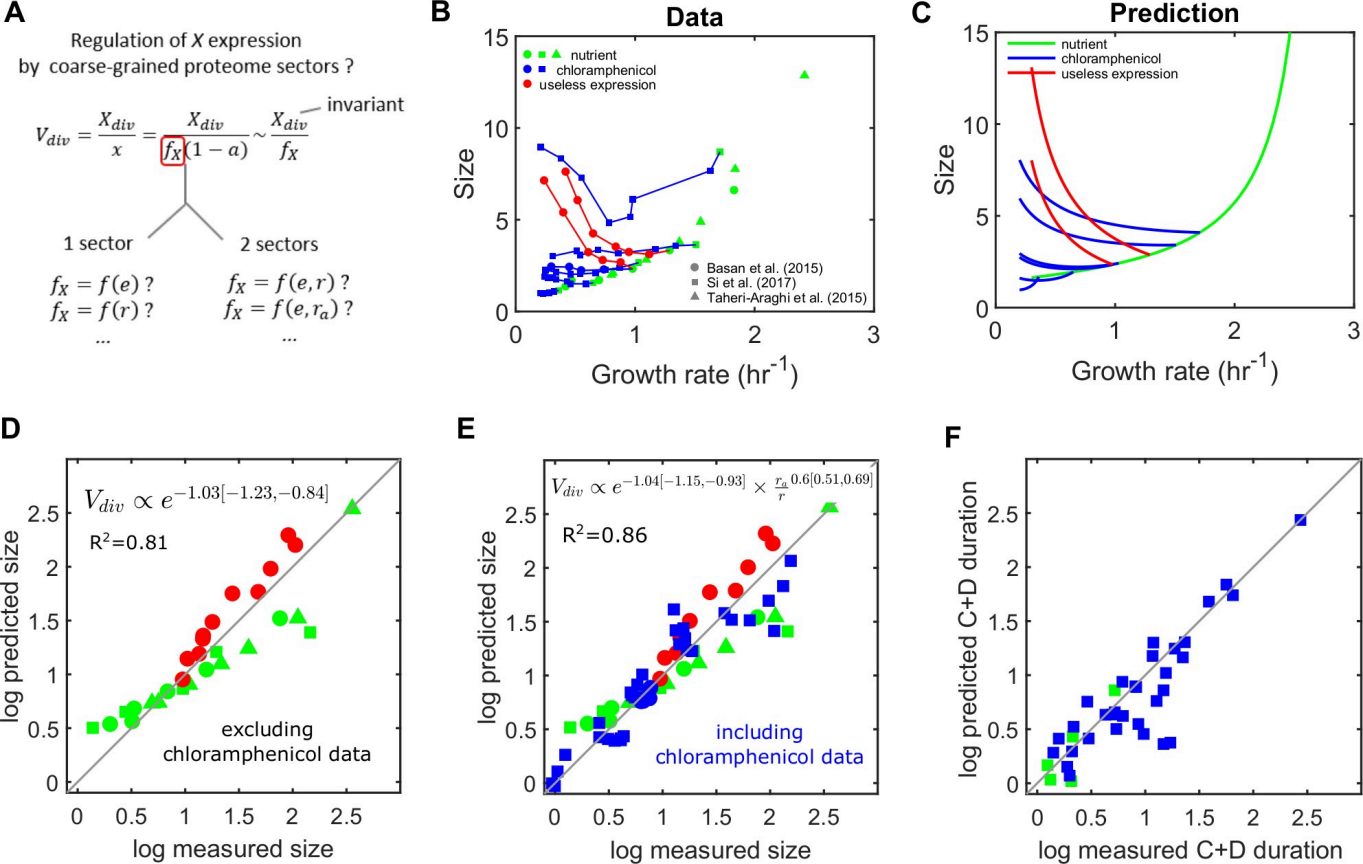
Regulation of division proteins by two proteome sectors quantitatively explain cell size across growth modulations. (A) Hypothesis stating that *X* expression could depend on the concentration of one or two coarse-grained proteome sectors. Here we assume that *X*_*div*_ is invariant. (B) Empirical relationship between cell size and growth rate for three types of growth rate modulation (nutrient quality, chloramphenicol-mediated translation inhibition and expression of useless protein). Data aggregated from three studies, Basan et al. (2015), Si et al. (2017) and Taheri-Araghi et al. (2015). A scaling factor for size was applied on Si et al. and Taheri-Araghi et al. data to make the nutrient modulation data of the three studies consistent (see Fig C in S1 Text). Different branches for useless expression and chloramphenicol indicate modulations at different nutrient quality. (C) Predicted relationship between cell size and growth rate when *X* expression depends on both *E* concentration and the fraction of active ribosomes. (D-F) Log-log plots comparing model predictions with experimental data. The natural log is used. (D) Regulation of *X* expression by *E* alone can explain size data for nutrient modulation and useless expression modulation (but not chloramphenicol-mediated translation inhibition, Fig D in S1 Text). (E) Among possible regulations of *X* by two coarse-grained quantities, regulation by *E* concentration and the fraction of active ribosomes explains size data for all three types of growth rate modulations (this regulation model was used for (C)). (F) Predicted C+D durations agree with measurements by Si et al. (2017). C+D is predicted by $C+D=\frac{log[V_{div}/S_{0}]}{\alpha }$, where α is the growth rate, *V*_*div*_ is the model-predicted size (3C and 3E) and *S*_0_ is a constant measured in Si et al. (2017).

Experimental data from previous studies shows a complex relationship between cell size and growth rate across the three types of growth modulation (Fig 3B, Fig C in S1 Text). While cell size increases with growth rate when nutrient quality is varied as posited by the second growth law, it decreases with the growth rate in response to overexpression of useless proteins. On the other hand, chloramphenicol-mediated translation inhibition leads to cell size increase or decrease depending on the nutrient quality of the medium. Basan and colleagues [29] proposed that a structural model of cell division requires the expression of the division protein *X* to follow the inverse of cell size assuming an invariant division threshold *X*_*div*_ (Fig 2B, bottom). We therefore asked whether these complex variations in cell size with the growth rate could be explained by the regulation of division proteins *X* by different coarse-grained proteome sectors via the allocation fraction *f*_*X*_ (Fig 3A).

We first considered a model whereby *f*_*X*_ follows the concentration of a single coarse-grained proteome sector. Interestingly, size measurements for both nutrient quality and useless protein expression growth rate modulations are explained if *f*_*X*_ is set to follow the concentration (noted *e*, not to confuse with the mathematical constant) of the metabolic sector *E* exclusively (Fig 3D). However, critically, this simple dependency cannot explain chloramphenicol-mediated translation inhibition data (Fig D in S1 Text). We therefore extended this analysis to dependencies of *f*_*X*_ on two coarse-grained proteome quantities and tested all pairwise combinations of the quantities *e*, *r*, *r*_*a*_ and $\frac{r_{a}}{r}$ (Fig E in S1 Text). We found that when *f*_*X*_ depends on both the *E* sector concentration *e* and the fraction of active ribosomes $\frac{r_{a}}{r}$, the observed relationship between size and growth rate is quantitatively captured for all types of growth rate modulation, including translation inhibition (Fig 3C and 3E). Interestingly, the exponent for the E sector concentration remained close to 1 when fitting two quantities instead of one and including chloramphenicol modulation (Fig 3D and 3E). This validates a consistent contribution of this sector across the three growth modulations. We note that the fitted exponent values are very close to 1 and -2/3 for ***e*** and $\frac{r_{a}}{r}$ respectively. Because *f*_*X*_ and cell size are inversely proportional, it then follows that cell size *V* scales with $e^{-1}\times ({\frac{r_{a}}{r})}^{2/3}$. Based on this we propose a novel size law formalized as:

$$V\propto e^{-1}\times ({\frac{r_{a}}{r})}^{2/3}$$

Our model assumes that in the absence of translation inhibition all ribosomes remain active ($\frac{r_{a}}{r}=1$). However, it has been observed that for nutrient-limited slow growth the active ribosome fraction is reduced [49]. Interestingly, using these experimental data instead of the model predictions improves the accuracy of the size law at slow growth rates (Fig F in S1 Text), providing further independent support for the proposed relationship between size and active ribosome fraction. The duration of the C+D period has been observed to have non-trivial variations (Si et al., 2017). We note that this size law can predict the duration of the C+D period (Fig 3F) when assuming that DNA replication occurs at a fixed condition-independent cell size (i.e. using the relationship V_div_ = S_0_ exp(α(C+D)), where α is the growth rate and S_0_ is the constant initiation size).

In summary, our minimalistic model quantitatively captures how both size and coarse-grained cell composition change with the growth rate for all three types of growth modulation with only two parameters (the two exponents of the *f*_*X*_ dependency on *e* and *r*_*a*_/*r*, those exponents being very close to 1 and -2/3 respectively). Therefore, *E*. *coli* cell size can be predicted from only two coarse-grained proteome quantities, metabolism sector concentration and the fraction of active ribosomes leading to the definition of a novel growth law.

### Emergence of ‘adder’ size homeostasis and cellular individuality in the presence of noise

**Fig 4:**
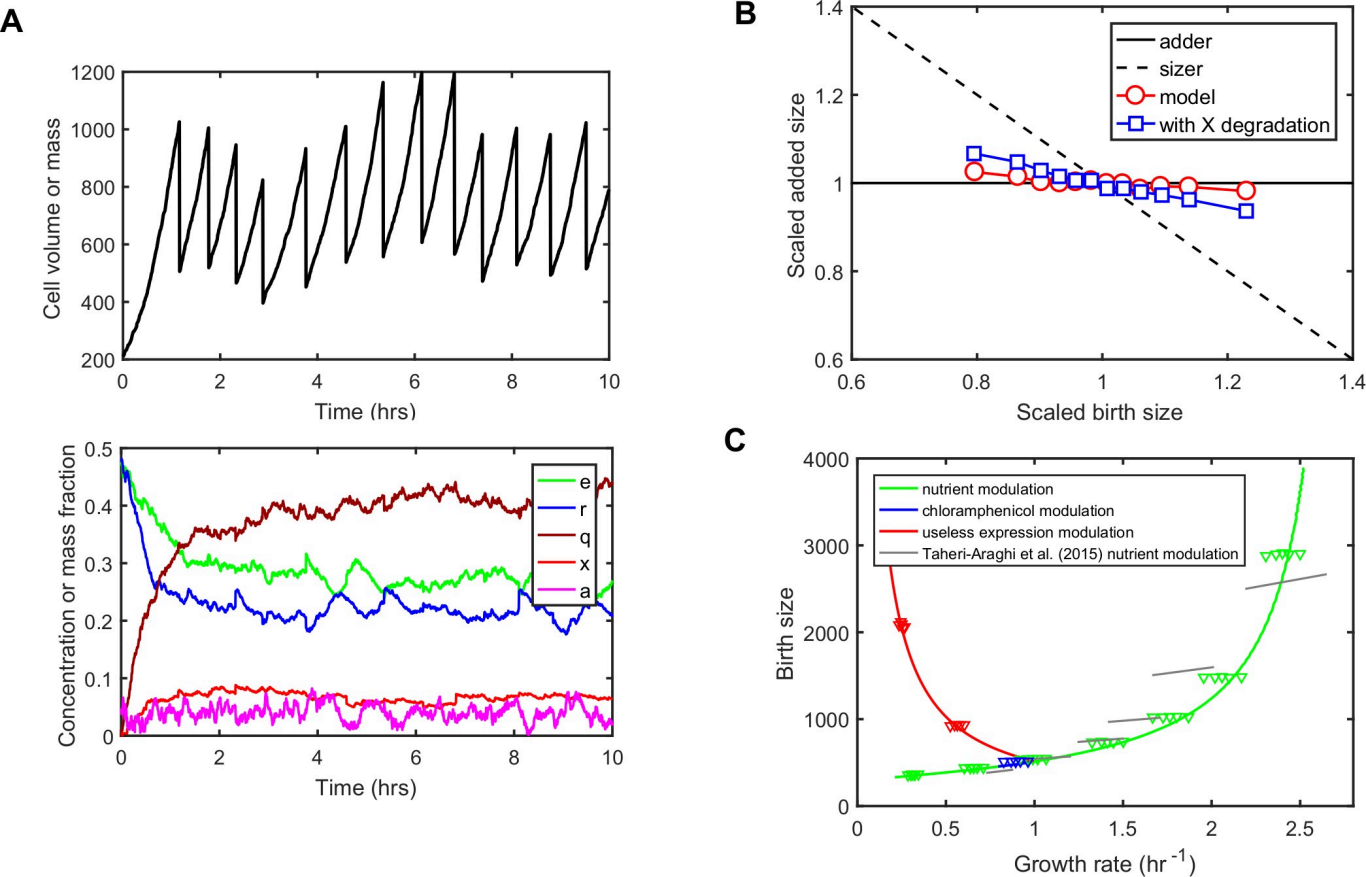
Emergence of ‘adder’ size homeostasis and cellular individuality in the presence of reaction noise. (A) Simulation of model dynamics with molecular noise (top: total cell size, bottom: concentrations of cellular components). At division, cellular components are randomly split between daughter cells, so the tracked daughter has a probability 1/2 of getting each mother cell component. Same parameters as in Fig 2A. The parameters *X*_*div*_ and $f_{X}^{scale}$ were chosen to obtain realistic cell-to-cell variability in size at birth and growth rate (Methods). (B) Model leads to near 'adder' size homeostasis. Average added size during one cell cycle as a function of birth cell size (via binning) is plotted. The very weak deviation towards ‘sizer’ can be explained by a residual correlation between size at birth and X count at birth due to the non-zero share of X in total size. A model variant where *X* is actively degraded at a constant rate and displaying a stronger deviation towards 'sizer' behavior is also shown. Other model variants exploring size homeostasis properties are discussed in Fig G in S1 Text. (C) Emergence of cellular individuality. Stochastic stimulations are performed for ten different growth conditions: seven nutrient qualities (green triangle groups), two useless expression strengths (red triangle groups) and one chloramphenicol (blue triangle groups). For each condition, cell cycles are binned by growth rate and the corresponding birth size is plotted. Continuous lines show the prediction of the deterministic model for the three growth rate modulations. Grey lines indicate experimental trends for different nutrient qualities extracted from mother machine data (Taheri-Araghi et al., 2015).

So far, we have neglected cell-to-cell variability and focused on predicting average cell composition, size and growth rate at steady-state as a function of growth conditions. However, isogenic cells growing in a constant environment show significant phenotypic variability, notably for global traits such as growth rate or division size [30,52–54]. Moreover, non-trivial correlations between single-cell traits are often observed and contain rich information about regulatory mechanisms [55,33,56,30,31,57,23,25]. Since our model is based on biochemical reactions between coarse-grained molecular components, dynamic cell-to-cell variability naturally emerges when adopting a stochastic interpretation of all reactions and random partitioning of components at division (Fig 4A).

In the deterministic model, the two parameters *X*_*div*_ (the number of division molecules *X* needed to trigger cell division) and $f_{X}^{scale}$ (the constant factor in $f_{X}=f_{X}^{scale}\times e{\times (\frac{r_{a}}{r})}^{-2/3}$) are only setting the size scale via their ratio (Fig 3A). In the presence of reaction noise, those parameters will now impact the extent of cell-to-cell variability in growth rate and in size at division. Even with this simple model of gene expression noise, we could find values of those two parameters that lead to reasonable estimates of both noise in growth rate and size at birth (Methods).

Stochastic simulations of our model lead to a near 'adder' size homeostasis, as observed experimentally (Fig 4B, red circles). Interestingly, the deviation towards a 'sizer' size homeostasis observed experimentally at very slow growth, [27] could also be explained by additional model assumptions (Fig G in S1 Text). These include the constant degradation of *X* during the cell cycle, which could dominate dilution at slow growth (Fig 4B, blue squares). We then explored the relationship between the size at birth and the growth rate of single cells for several conditions across the three types of growth rate modulation (Fig 4C, triangles). This relationship at the level of single cells is known to deviate from the one connecting average size at birth and average growth rate when conditions are varied [30,31]. These deviations were qualitatively captured by our stochastic model (Fig 4C, compare grey lines and green triangle groups).

In summary, our minimalistic coarse-grained model generates realistic cell-to-cell variability in growth rate and cell size, as well as realistic correlations between added size and birth size ('adder' size homeostasis) and between growth rate and birth size.

## Discussion

Our aim in this work was to provide a mechanistic understanding of the variations in cell size across growth conditions recently observed in E. coli. To this end, we have proposed a minimalistic whole-cell coarse-grained model of E. coli physiology to predict the relationship between growth rate, cell composition, ribosome activity and cell size for three different types of growth rate modulation (Fig 1A and Fig 2). Our model builds on and unifies previous efforts to understand proteome allocation [15,13,10,16,20] and division control [48,29,30,58]. While other theoretical models [13,10,16,20] have also captured proteome allocation data, our model stands out because: 1) it includes ribosome activity and captures more data; 2) it is holistic with respect to cell composition and cell size and does not a priori neglect the mass fraction of protein precursors; and 3) it is not based on optimization of growth rate but rather a positive regulation of ribosome synthesis by precursors. Moreover, despite its simplicity and low number of free parameters (only the exponents in the size law were fitted and obtained to be close to -1 and 2/3 respectively; the other four parameters were constrained by previous studies), our model quantitatively reproduces experimental data on proteome allocation (Fig 1C and 1D), ribosome activity (Fig 1E and 1F) and average cell size (Fig 3C and 3E) for the three types of growth rate modulation (change of nutrient quality, chloramphenicol-mediated ribosome inactivation and expression of useless proteins). Our model therefore compares favourably to existing coarse-grained models in terms of number of parameters (for instance, the pioneering model by Weisse and colleagues [16] has more than 10 parameters and is only focused on data from Scott and colleagues [13]). In addition, when reaction noise is included in the model, experimental observations such as the *adder* principle of size homeostasis and non-trivial correlations between single-cell cellular growth rate and cell size are predicted (Fig 4).

A remarkable result of our study is the emergence of a novel size law related to the second growth law. This law states that only one coarse-grained quantity characterizing cell composition is sufficient to predict quantitatively cell size changes in response to nutrient quality and to over-expression of a useless protein (Fig 3C and 3E, Fig D in S1 Text). A second coarse-grained quantity is required for prediction of cell size when translation is inhibited. Those quantities are the mass fraction (or equivalently, concentration) of metabolic proteins (*e* in the model) and the fraction of total ribosomes that are active (*r*_*a*_/*r*), respectively. Strikingly, best fit is robustly obtained for exponent values close to ~−1 for the metabolic protein concentration and ~2/3 for the fraction of active ribosomes. This results in a ‘size law’ stating: $V\propto e^{-1}\times ({\frac{r_{a}}{r})}^{2/3}$. We did not impose constraints on the values of exponents when fitting size data, the fact that fitted exponents are close to such particular values is therefore remarkable and suggests a fundamental underlying physical mechanism. Also, as reported in Fig F in S1 Text, the quality of the size predictions for nutrient modulation data at slow growth increases when the empirical active fractions of ribosomes are accounted for (below 1 at very slow growth, while our model assumes 1). This further validates the contribution of the active fraction of ribosomes to the law. In the absence of translation inhibition and at intermediate to fast growth the fraction of active ribosomes is close to 1 (Dai et al. 2016), therefore the law reduces to the simple form *V*∝*e*^−1^. This relationship has been observed before for modulation of growth in the absence of translation inhibition [30]. Basan and colleagues [29] also noted that if the growth rate dependence of *X* protein follows the one of constitutively expressed proteins for modulation by either nutrient quality or over-expression of useless proteins, then their size data could be explained. Because expression of the metabolic sector has the same growth rate dependence as constitutive expression upon nutrient modulation [13], our results agree with those previous observations. However, neither studies were able to explain cell size measurements upon translation inhibition with chloramphenicol. Basan and colleagues [29] opted for a conservative approach designed to uncover only one structural protein and which relied on condition-independent division thresholds. Our approach, on the other hand, is less restrictive and integrate proteome allocation with cell size data leveraging all predictions of our coarse-grained model, including different proteome sectors and ribosome activities. This allowed to uncover the role for ribosome activity in setting cell size and to capture experimental data from changing conditions including translation inhibition.

Our ‘size law’ is very different in nature from the second growth law stating that *V*∝exp[*α*(*C*+*D*)], where *α* is the growth rate, *C* the duration of DNA replication and *D* the duration between end of DNA replication and cell division. By construction, the second growth law links size at division to the control of DNA replication by size [26,27,44]. It predicts a simple exponential dependency with growth rate for nutrient quality modulation because those three quantities are largely independent of growth rate. However, while average size per origin at replication initiation is invariant across a wide range of growth conditions, both *C* and *D* durations change with growth conditions in complex ways [28]. Therefore, the second growth law is replication-initiation-centric and of limited use to make predictions on cell size at division outside of the canonical type of growth rate modulation (change in nutrient quality). In contrast, the ‘size law’ which we propose here links coarse-grained cell composition to cell size directly; therefore, by assuming the second growth law, we can also explain the observed variation in the *C* and *D* durations at least across three different types of growth rate modulation. This law can generate directly testable predictions, such as cell size and C+D durations for growth modulations involving both over-expression of useless proteins and translation inhibition by chloramphenicol.

In agreement with our results, recent studies contradict the replication-initiation-centric view of cell size control [44]. Based on single-cell correlation statistics, Micali and colleagues [46] propose that two concurrent processes, each controlling DNA replication and division, co-determine the effective size at division. Furthermore, Si and colleagues [47] experimentally demonstrated that two independent ‘adder’ homeostasis mechanisms are in place for DNA replication initiation and cell division. This does not mean that cellular constraints related to DNA replication are not affecting average cell size at division. Yet it indicates that cell division is not enslaved to the process controlling DNA replication initiation. Also, recent experiments reported different amounts of DNA in newborn cells, suggesting the chromosome is not involved in size homeostasis or responsible for the adder behavior [59]. Here we find that homeostatic properties at the level of cell division appear consistent with the structural model of division control. As shown in Fig 4, the structural model implemented at the single cell level recovers ‘adder’ size control [58], deviation towards ‘sizer’ [27] in some parameter regimes (Fig G in S1 Text) and non-trivial deviations from growth law at the single cell level [30,31]. We note that as suggested by a recent model [46], there could be still a role for DNA-replication or segregation in division control, for example when these processes are slowed down [28,35].

In our model, the quantitative relationship between cell composition and cell size is explained via a dependence of the allocation fraction of division proteins *X* in *e* and $\frac{r_{a}}{r}$ (Fig 3A), but this relationship is also valid in itself as a phenomenological observation (Fig 3C). Thus, the observed growth law is independent of the validity of the structural model and our whole-cell coarse-grained model. In addition, although the total ***X*** protein fraction is likely to be negligible, as it is commonly the case for cell cycle regulators, it should still follow the regulation of the major proteome fractions in the cell. What could explain the quantitative relationship between the coarse-grained quantities *e* and $\frac{r_{a}}{r}$ and cell size? Under the structural model of division control, assuming division threshold *X*_*div*_ is invariant across conditions (Fig 3A), it would imply that the *X* allocation fraction *f*_*X*_ scales with *e* and the inverse of $({\frac{r_{a}}{r})}^{2/3}$. In such case, the dependency on the active ribosome fraction could for example reflect chloramphenicol-mediated alteration of *X* expression, such as operon polarity via premature transcription termination [60]. However, under the same assumption that *X*_*div*_ is invariant, Basan and colleagues [29] could not identify in proteomics data candidate proteins whose proteome fraction is behaving quantitatively as you would expect for the factor *X*, that is as the inverse of their cell size data. This result suggests that there may be no single protein triggering division when reaching a fixed, condition-independent amount. An alternative possibility consistent with our results is that *X* follows exactly the relative abundance of metabolic proteins *E*, while the division threshold *X*_*div*_ scales with the fraction of active ribosomes to the power 2/3. Other constraints could explain a condition-dependent threshold *X*_*div*_ such as the cell geometry [45]. A recent study reported that rod-shaped bacteria like *E*. *coli* maintain an approximately constant aspect ratio across various growth conditions, resulting in the cell surface area scaling as the cell volume to the power 2/3 [61].

It is also possible that the resource allocation that maximizes growth in a given condition imposes a ratio between surface and cytoplasmic proteins that constrains the surface-to-volume ratio (i.e. the cell width for rod-shaped organisms). In turn, depending on the mechanism ‘counting’ the absolute amount of *X* molecules, the threshold can depend on cell width. In the context of a width-dependent threshold, as suggested by several studies *FtsZ* is an attractive candidate as the sole *X* factor [62,45,63,47]. Another mechanism involving geometrical constraints is the recent observation that ribosomes are spatially organized via nucleoid exclusion and that nucleoid size scales with cell size [64].

In this work, we have tried to explain the mechanistic origin of size regulation across growth conditions. Whether this is an evolved trait that optimizes fitness is a more difficult question. While size changes due to expression of useless proteins or translation inhibition by drugs may not be an evolved trait, the increased size with nutrient modulation of growth rate seems to be a universal property of microbes, which makes it likely to be an evolved property of unicellular systems [65,66]. In a recent *in silico* study, we showed that increased size at fast growth prevents an increase in gene expression noise for proteins with decreasing concentrations [67]. Metabolic proteins *E* are such proteins, and noise in their expression is likely detrimental to fitness. Interestingly, the inverse relationship between cell size and metabolic protein concentration *e* found in this study implies that the total number of metabolic proteins is kept constant across growth conditions. This raises the possibility that the evolution of size regulation in response to growth conditions has been driven by selective pressures acting on metabolic protein expression noise rather than on size directly. Testing fitness benefit of size regulation is difficult [63,68] but should ultimately shed light on the constraints that shaped its evolution.

Modelling cellular systems by considering the cellular context within which they operate is fundamental to a full mechanistic understanding of their function. Modelling has shown that function of simple synthetic networks can be affected by cell cycle and cell growth, when cell physiology and resource allocation is taken into account [6,16,69,67]. The current study is a first attempt at integrating coarse-grained models of cell physiology with simple models of division control, which has revealed a novel size law providing a link between cell size and cellular resource allocation. Overall, our study highlights the importance of systematic integration of whole-cell physiological models in the study of natural and synthetic cellular systems.

## Methods

### Model reactions

Our model considers the following reactions between coarse-grained cell components:

$\emptyset \overset{kE}{\to }A$ (nutrient import and transformation into protein precursors *A*)

$A\overset{f_{E}\sigma R_{a}\frac{a}{a+a_{sat}}}{\to }E$ (synthesis of *E* proteins)

$A\overset{f_{R}\sigma R_{a}\frac{a}{a+a_{sat}}}{\to }R_{a}$ (synthesis of active *R* proteins)

$R_{a}\overset{k_{on}^{cm}R_{a}}{\to }R_{i}$ (inactivation of active *R* proteins via chloramphenicol binding)

$R_{i}\overset{k_{off}^{cm}R_{i}}{\to }R_{a}$ (re-activation of chloramphenicol-inactivated *R* proteins via chloramphenicol unbinding)

$A\overset{f_{Q}\sigma R_{a}\frac{a}{a+a_{sat}}}{\to }Q$ (synthesis of *Q* proteins)

$A\overset{f_{X}\sigma R_{a}\frac{a}{a+a_{sat}}}{\to }X$ (synthesis of *X* proteins)

$A\overset{f_{U}\sigma R_{a}\frac{a}{a+a_{sat}}}{\to }U$ (synthesis of *U* proteins)

Where the cell volume is *V* = *E*+*R*+*Q*+*X*+*U*+*A* (with *R* = *R*_*a*_+*R*_*i*_), the protein synthesis allocation parameters (*f*_*E*_,*f*_*R*_,…) respect the constraint *f*_*E*_+*f*_*R*_+*f*_*Q*_+*f*_*X*_+*f*_*U*_ = 1, and $a=\frac{A}{V}$ (we will also note *e*, *r*, *q*, *x*, *u*, … the concentrations *E/V*, *R/V*, etc…). The parameter *k* is the medium nutrient quality, $k_{on}^{cm}$ is the chloramphenicol-imposed inactivation rate of ribosomes and *f*_*U*_ is the imposed allocation fraction of useless proteins. Those three parameters represent the three types of growth rate modulations that we consider. The parameter *σ* is the maximal rate of protein synthesis by one ribosome and *a*_*sat*_ is the enzymatic saturation constant of ribosomes. The parameter $k_{off}^{cm}$ is the dissociation rate of chloramphenicol-ribosome complexes. Note that all protein synthesis reactions conserve mass, so that volume growth equals nutrient import ($\frac{dV}{dt}=kE$ as seen below).

### Differential equations

A deterministic interpretation of these reactions leads to the following set of differential equations for absolute amounts of coarse-grained cell components (whose absolute mass can be evaluated to approximately 0.15 femtograms, given typical values of Xdiv and fX considered here and the typical mass of a cell):

$$\frac{dA}{dt}=kE-\sigma R_{a}\frac{a}{a+a_{sat}}$$

$$\frac{dE}{dt}=f_{E}\sigma R_{a}\frac{a}{a+a_{sat}}$$

$$\frac{dR_{a}}{dt}=f_{R}\sigma R_{a}\frac{a}{a+a_{sat}}-k_{on}^{cm}\;R_{a}+k_{off}^{cm}\;R_{i}$$

$$\frac{dQ}{dt}=f_{Q}\sigma R_{a}\frac{a}{a+a_{sat}}$$

$$\frac{dX}{dt}=f_{X}\sigma R_{a}\frac{a}{a+a_{sat}}$$

$$\frac{dU}{dt}=f_{U}\sigma R_{a}\frac{a}{a+a_{sat}}$$

$$\frac{dR_{i}}{dt}=k_{on}^{cm}\;R_{a}-k_{off}^{cm}\;R_{i}$$

It can be seen that the right-hand side of those equations sum up to *kE*. The above differential equations can be also written in terms of concentrations, noticing that $\frac{dV}{dt}=kE=k\;e\;V$:

$$\frac{da}{dt}=ke-\sigma r_{a}\frac{a}{a+a_{sat}}-kea$$

$$\frac{de}{dt}=f_{E}\sigma r_{a}\frac{a}{a+a_{sat}}-ke^{2}$$

$$\frac{dr_{a}}{dt}=f_{R}\sigma r_{a}\frac{a}{a+a_{sat}}-k_{on}^{cm}r_{a}+k_{off}^{cm}r_{i}-ker_{a}$$

$$\frac{dq}{dt}=f_{Q}\sigma r_{a}\frac{a}{a+a_{sat}}-keq$$

$$\frac{dx}{dt}=f_{X}\sigma r_{a}\frac{a}{a+a_{sat}}-kex$$

$$\frac{du}{dt}=f_{U}\sigma r_{a}\frac{a}{a+a_{sat}}-keu$$

$$\frac{dr_{i}}{dt}=f_{R}\sigma r_{a}\frac{a}{a+a_{sat}}+k_{on}^{cm}r_{a}-k_{off}^{cm}r_{i}-ker_{i}$$

In the above equations, the last terms represent dilution by cell growth (*k e*). Because concentrations are conserved at division, their dynamics and steady-state values are independent of the division threshold *X*_*div*_.

### Steady-state growth

In this model, steady-state growth (or balanced growth, i.e. steady concentrations of coarse-grained cell components) is necessarily exponential at rate *α* = *k e*, because $\frac{dV}{dt}=kE=k\;e\;V$. By setting the time derivatives to zero, we obtain the following equations constraining the steady-state cell composition (*e*,*r*_*a*_,*r*_*i*_,*q*,*x*,*u*,*a*):

$$ke(1-a)=\sigma r_{a}\frac{a}{a+a_{sat}}$$

$$ke^{2}=f_{E}\sigma r_{a}\frac{a}{a+a_{sat}}$$

$$ker_{a}=f_{R}\sigma r_{a}\frac{a}{a+a_{sat}}-k_{on}^{cm}\;r_{a}+k_{off}^{cm}\;r_{i}$$

$$keq=f_{Q}\sigma r_{a}\frac{a}{a+a_{sat}}$$

$$kex=f_{X}\sigma r_{a}\frac{a}{a+a_{sat}}$$

$$keu=f_{U}\sigma r_{a}\frac{a}{a+a_{sat}}$$

$$ker_{i}=k_{on}^{cm}\;r_{a}-k_{off}^{cm}\;r_{i}$$

The first equation gives another expression for the growth rate: $\alpha =ke=\sigma \frac{r_{a}}{1-a}\frac{a}{a+a_{sat}}$, where the factor $\frac{r_{a}}{1-a}$ is in fact the proteome fraction of active ribosomes *φ*_*Ra*_. It also reflects the balance between the rate of nutrient import and transformation into precursors and the rate of total protein synthesis in the context of dilution imposed by mass density homeostasis.

Combining the first equation with every other enables expressing protein concentrations as a function of (1−*a*):

$$e=f_{E}(1-a),\;r=f_{R}(1-a),\;q=f_{Q}(1-a)\;\mathrm{and}\;x=f_{X}(1-a)$$

This is intuitive: a proteome sector concentration is equal to its allocation fraction times the total protein concentration 1−*a*. We also see that steady-state proteome fractions *φ* are equal to allocation fractions *f*.

Noting that $\frac{r_{i}}{r_{a}}=\frac{k_{on}^{cm}}{k_{off}^{cm}+kf_{E}(1-a)}$ (from the steady-state equation for *r*_*i*_ together with *e* = *f*_*E*_(1−*a*)) and that $f_{R}\;\sigma \frac{a}{a+a_{sat}}=k_{on}^{cm}+kf_{E}(1-a)-k_{off}^{cm}\frac{r_{i}}{r_{a}}$ (from the steady-state equation for *r*_*a*_ divided by *r*_*a*_) leads to an equation for *a* by substituting the first expression in the second:

$$f_{R}\;\sigma \frac{a}{a+a_{sat}}=kf_{E}(1-a)\frac{kf_{E}(1-a)+k_{on}^{cm}+k_{off}^{cm}}{{kf_{E}(1-a)+k}_{off}^{cm}}$$

Solving this equation for *a* allows to compute the steady-state solution for fixed allocation fractions *f*_*E*_, *f*_*R*_, *f*_*Q*_, *f*_*X*_ and *f*_*U*_.

### Dynamic regulation of allocation fractions

Dai and colleagues [49] found that the translation elongation rate displays a Michaelis-Menten like dependence with the RNA to protein ratio (which is proportional to the total ribosome proteome fraction) across nutrient conditions and chloramphenicol-mediated translation inhibition. A simple way to reproduce these findings with our model is to assume that the allocation fraction *f*_*R*_ is regulated by the concentration of precursors via direct proportionality: *f*_*R*_ = *δ*∙*a*. Indeed, we would then have $\frac{a}{a+a_{sat}}=\frac{\delta^{-1}f_{R}}{\delta^{-1}f_{R}+a_{sat}}=\frac{f_{R}}{f_{R}+\delta a_{sat}}$. Note that such regulation is a form of supply-driven activation [10].

Therefore, our model for how allocations fractions are dynamically set is as follows:

- *f*_*Q*_, *f*_*U*_, *f*_*X*_ are constants (*f*_*Q*_ is also invariant across conditions)
- *f*_*R*_ = *δ*∙*a* (or 1−*f*_*Q*_−*f*_*U*_−*f*_*X*_ if *δ*∙*a*>1−*f*_*Q*_−*f*_*U*_−*f*_*X*_)
- *f*_*E*_ = 1−*f*_*R*_−*f*_*Q*_−*f*_*U*_−*f*_*X*_

The ordinary differential equations (ODEs) presented above with such dynamically changing allocations fractions are used to simulate Fig 2A, together with the halving of all amounts at division, which is triggered when *X* = *X*_*div*_. The integration of the ODEs together with the handling of division events is natively supported by MATLAB ODEs solving suite. We used ode45 with default tolerances. A pseudo-code description of the simulation is therefore:

- Initialize amounts of A, E, RA, RI, Q, U, and X
- Integrate the ODEs (using parameters *δ*, *a*_*sat*_ = *K*/*δ*, *f*_*Q*_, *f*_*X*_, $k_{off}^{cm}$, k, $k_{on}^{cm},$ *f*_*U*_) until X reaches the division threshold Xdiv
- Divide all amounts by 2 and repeat previous step

### Steady-state proteome fractions for the regulation model

By substituting *f*_*R*_ = *δ*∙*a* in the steady-state growth equations above, we obtain the following equation if we further assume that *a*≪1 (i.e. *δ* is large enough):

$$f_{R}\sigma \frac{f_{R}}{f_{R}+K}=kf_{E}\left(1+\frac{k_{on}^{cm}}{k_{off}^{cm}+kf_{E}}\right),\;\mathrm{where}\;K=\delta a_{sat}$$

Because *f*_*E*_ = 1−*f*_*R*_−*f*_*Q*_−*f*_*U*_−*f*_*X*_, this can be solved numerically by scalar optimization on *f*_*R*_.

This steady-state approximation was used for the results presented in Figs 1 and 3. However, when simulating the dynamic model (Figs 2 and 4), a value for *δ* has to be chosen, and the allocation fraction *f*_*X*_ has to be non-zero. We chose *δ* = 5 and we subtracted *f*_*X*_ from *f*_*Q*_ to ensure *f*_*Q*_+*f*_*X*_ = 0.5. We show in Fig A in S1 Text the validity of the *a*≪1 approximation.

### Optimality of proteome allocation fractions

Above we considered the case where the allocation fractions are dictated by a supply-driven activation of ribosome synthesis. For given growth conditions, one could also search for the proteome allocation fractions that maximize growth rate (under the constraints of a fixed, condition-independent allocation fraction to housekeeping proteins *f*_*Q*_, an imposed allocation fraction to useless proteins *f*_*U*_, and under the assumption that *f*_*X*_≪1).

This is achieved by enumerating all possible allocations by varying *f*_*R*_ in between 0 and 1−*f*_*Q*_−*f*_*U*_, and computing the growth rate of the corresponding steady-state (we use the bounded scalar optimization *MATLAB* function *fminbnd*).

Such optimality assumptions are sometimes made in previous work, such as in Molenaar et al. (2009).

### Model parameterization

Cell composition data is taken from (Scott et al. 2010) and (Dai et al. 2016) [13,49]. It consists of steady-state ribosome proteome fractions for a combination of nutrient conditions and chloramphenicol concentrations (measured via the RNA/protein ratio). The dataset from Dai et al. (2016) also provides translation elongation rate and estimated fraction of ribosomes that are active. Following Scott and colleagues [13], we used a conversion factor of 0.76 to convert RNA/protein ratios into proteome fractions of extended ribosomes. For consistency, we also used the notion of extended ribosomes (1.67 times larger than a single ribosome, Scott et al., 2010) to convert the maximal elongation rate of 22 aa/s measured by Dai and colleagues [49] into a maximal rate of protein synthesis (*σ* in our model). Also, based on maximal ribosome proteome fractions observed by Scott and colleagues, we set the value of *f*_*Q*_ to 0.5. Finally, we used the value of the unbinding constant $k_{off}^{cm}$ for chloramphenicol-ribosome complexes given in [49]. Altogether, we obtained the following parameterization from those studies:

$$\sigma =\frac{1}{\frac{7336\;aa*1.67}{22\;aa.s^{-1}}}s^{-1}=6.46\;{hr}^{-1}$$

K=0.11*0.76=0.0836

$$f_{Q}=0.5$$

$$k_{off}^{cm}=5.04\;{hr}^{-1}$$

### Finding regulation of *f*_*X*_ expression explaining cell size data across types of growth rate modulation

The structural model of cell division links the steady-state cell composition and cell division size via the concentration *x* of *X* proteins: $V_{div}=\frac{X_{div}}{x}=X_{div}\frac{1}{f_{X}(1-a)}$, which approximates to $\frac{X_{div}}{f_{X}}$ under the low precursor concentration assumption.

To search for regulation of *f*_*X*_ explaining cell size data across the three types of growth rate modulations, we considered the following form of $f_{X}:f_{X}\propto c_{1}^{\beta_{1}}c_{2}^{\beta_{2}}$ where *c*_1,2_ are quantities depending on the coarse-grained cell composition and *β*_1,2_ are exponents. This functional form was chosen for two reasons. First it the simplest form beyond regulation only by a single factor. Second, regulation of gene expression by several types of molecules (such as transcription factors and co-factors) is likely to be multiplicative.

We considered the following coarse-grained quantities: coarse-grained concentrations *e*, *r*, *r*_*a*_ and the fraction of active ribosomes $\frac{r_{a}}{r}$ (not to confuse with the mass fraction of active ribosomes *r*_*a*_ or the proteome fraction of active ribosomes $f_{RA}=\frac{r-r_{i}}{1-a}$).

Fitting was performed using multilinear regression on the logarithm of cell size data using *MATLAB* function *regress*. 95% confidence intervals on exponents were provided by the *regress* function. Cell size data between different datasets was normalized as detailed in Fig C in S1 Text.

To estimate the *C+D* duration directly from our size estimate, we use the value of ‘unit cell’ or DNA replication initiation volume from (Si et al. 2017) (*S*_0_ = 0.28 *μm*^3^, converted to the normalized size scale described in Fig C in S1 Text).

### Stochastic model

The stochastic version of the model was simulated using the *Gillespie* algorithm and in a mother machine setting. When *X* reaches the division threshold *X*_*div*_, each coarse-grained molecule is kept in the daughter cell that we keep simulating with probability ½. Note that we are modeling protein synthesis with a single reaction for each sector, hence abstracting away transcription and mRNA degradation. Therefore, there are two sources of noise in the model: stochasticity in reactions and partitioning of molecules at cell division.

### Code availability

All the code for solving model steady-state, simulating model dynamics, parameter fitting, etc. as well as the scripts generating all figures is available on GitHub. It is written in MATLAB except the stochastic simulation code written in C++. All simulations and calculations were performed on a DELL laptop (Precision 5520, Windows 10). MATLAB version 2016b was used and only built-in MATLAB functions were used.

## Supporting information

S1 Text Supporting information for “a bacterial size law revealed by a coarse-grained model of cell physiology”. (PDF)
